# COUP-TFII Mediates Progesterone Regulation of Uterine Implantation by Controlling ER Activity

**DOI:** 10.1371/journal.pgen.0030102

**Published:** 2007-06-22

**Authors:** Isao Kurihara, Dong-Kee Lee, Fabrice G Petit, Jaewook Jeong, Kevin Lee, John P Lydon, Francesco J DeMayo, Ming-Jer Tsai, Sophia Y Tsai

**Affiliations:** 1 Department of Molecular and Cellular Biology, Baylor College of Medicine, Houston, Texas, United States of America; 2 Program of Developmental Biology, Baylor College of Medicine, Houston, Texas, United States of America; Stanford University School of Medicine, United States of America

## Abstract

Progesterone and estrogen are critical regulators of uterine receptivity. To facilitate uterine remodeling for embryo attachment, estrogen activity in the uterine epithelia is attenuated by progesterone; however, the molecular mechanism by which this occurs is poorly defined. COUP-TFII (chicken ovalbumin upstream promoter transcription factor II; also known as NR2F2), a member of the nuclear receptor superfamily, is highly expressed in the uterine stroma and its expression is regulated by the progesterone–Indian hedgehog–Patched signaling axis that emanates from the epithelium. To further assess COUP-TFII uterine function, a conditional *COUP-TFII* knockout mouse was generated. This mutant mouse is infertile due to implantation failure, in which both embryo attachment and uterine decidualization are impaired. Using this animal model, we have identified a novel genetic pathway in which BMP2 lies downstream of COUP-TFII. Epithelial progesterone-induced Indian hedgehog regulates stromal COUP-TFII, which in turn controls BMP2 to allow decidualization to manifest in vivo. Interestingly, enhanced epithelial estrogen activity, which impedes maturation of the receptive uterus, was clearly observed in the absence of stromal-derived COUP-TFII. This finding is consistent with the notion that progesterone exerts its control of implantation through uterine epithelial-stromal cross-talk and reveals that stromal-derived COUP-TFII is an essential mediator of this complex cross-communication pathway. This finding also provides a new signaling paradigm for steroid hormone regulation in female reproductive biology, with attendant implications for furthering our understanding of the molecular mechanisms that underlie dysregulation of hormonal signaling in such human reproductive disorders as endometriosis and endometrial cancer.

## Author Summary

Pregnancy is established and maintained through a series of precisely choreographed cellular and molecular events that are controlled by two sex hormones, estrogen and progesterone. Both hormones exert their actions through their distinct nuclear receptors. During the peri-implantation period, estrogen activity is attenuated by progesterone to facilitate epithelial remodeling and embryo attachment, but the detailed molecular mechanism of how this process is achieved remains largely undefined. COUP-TFII (chicken ovalbumin upstream promoter transcription factor II; also known as NR2F2), a member of the nuclear receptor superfamily, is highly expressed in the uterine stroma, and its expression is controlled by progesterone–Indian hedgehog–Patched signaling from the epithelium to the stroma. To assess the uterine function of COUP-TFII, uterine-specific *COUP-TFII* knockout mice were generated. These mutant mice are infertile due to failure of implantation. We identified a novel genetic pathway in which the epithelial Ihh regulates the stroma COUP-TFII to control BMP2 and regulates decidualization. Interestingly, enhanced epithelial estrogen activity, which impedes the maturation of receptive uterus, was clearly noted in the absence of COUP-TFII. This finding reveals that COUP-TFII plays a critical role in maintaining the balance between estrogen and progesterone activities to establish proper implantation. This finding also provides new insights into women's health care associated with uncontrolled estrogen activity, such as breast cancer and endometriosis.

## Introduction

Establishment of uterine receptivity is mandatory for successful embryo apposition, attachment, and implantation; failure to manifest this uterine state is an underlying cause of most pregnancy failures in women. A multitude of signaling molecules have been shown to play key roles in the elaboration of this uterine response through mesenchymal–epithelial interaction. Among numerous factors involved in these primary events of pregnancy, two steroid hormone receptors, progesterone receptor (PR) and estrogen receptor (ER), and their cognate ligands, undoubtedly play central roles in this biological process [1–3]. Although estrogen activity is essential for an integrated uterine response, it has been shown that excessive estrogen activity can prematurely close the implantation window [4], suggesting that estrogen activity is tightly controlled during the peri-implantation period to allow normal development of the receptive uterus. Importantly, progesterone is known to attenuate estrogen-induced gene expression in uterine epithelial cells [5]. Intriguingly, this suppression is mediated by stromal progesterone receptors [6,7], suggesting that the coordinated action of estrogen and progesterone depends on crosstalk between the epithelial and stromal compartments of the uterus. Although the inhibitory effect of progesterone on epithelial estrogen activity has been described [6,7], the mechanism by which progesterone suppresses estrogen action remains poorly defined.

Lydon et al have shown that female PR-null mice are infertile [8]. The expression of *Indian hedgehog (Ihh),* a gene highly expressed in the uterine epithelium, is greatly reduced in these null mutants, indicating that *Ihh* is a downstream target of the progesterone receptor [9]. To understand the role of Ihh in reproduction, conditional null mutant mice of *Ihh* were generated [10]. These mutants exhibit defects in both implantation and decidualization, indicating that epithelial Ihh regulates the decidual response through Patched/Smoothened (Ptch/Smo) signaling in the stroma. Recently, it has been shown that COUP-TFII (chicken ovalbumin upstream promoter transcription factor II; also known as NR2F2) is a downstream target of Ihh in the uterine tissue [9,10]. COUP-TFII is highly expressed in the uterine stromal compartment, and its expression is significantly reduced in the *Ihh* mutant mice, suggesting that COUP-TFII might mediate the effects of Ihh signaling in the uterine stroma [10]. This notion is consistent with our previously findings that COUP-TFII is a downstream target of sonic hedgehog (Shh) signaling [9–13] and conditional ablation of *COUP-TFII* in the foregut mesenchyme resembles *Shh*-null mutant phenotypes [14].

COUP-TFII belongs to the orphan nuclear receptor superfamily, and has been well characterized [15,16]. Genetic ablation of *COUP-TFII* results in early embryonic lethality due to cardiovascular defects [17]. *COUP-TFII* heterozygous female mice have shown significant reduced fecundity, which is attributed to ovarian and uterine defects [18], but the uterine-specific function of COUP-TFII remains largely undefined. We have recently established a *COUP-TFII^flox/flox^* mouse model in order to generate tissue- or cell-lineage–specific knockouts of *COUP-TFII.* Phenotypes exhibited by conditional mutants lacking *COUP-TFII* in endothelial cells [19], limbs [20], stomach [14], or diaphragm [21] are all consistent with the notion that COUP-TFII might play an important role in reciprocal epithelial–mesenchymal cellular cross-talk.

To define *COUP-TFII* uterine function, we have ablated *COUP-TFII* specifically in cell lineages of the uterus that express PR by crossing *COUP-TFII^flox/flox^* with *PR-Cre* knockin mice [22]. Ablation of *COUP-TFII* in the uterine stroma results in decidualization failure, resembling the conditional ablation of *Ihh.* In addition, we showed that the expression of bone morphogenetic protein 2 (BMP2) is greatly reduced in the *COUP-TFII* mutants, and that reintroduction of BMP2 into uterine horn rescues the decidualization defects. Thus, we established a genetic pathway in which progesterone receptor regulates *Ihh*, which in turn regulates COUP-TFII through Ptch/Smo signaling, and finally, COUP-TFII regulates BMP2 to confer decidualization in the uterus. Surprisingly, we also found that ER activity and target gene expression in the uterine epithelial cells are markedly elevated in conditional *COUP-TFII* knockout mice, which alters the window of receptivity and affects embryo attachment and implantation. Since stromal PR has been implicated to suppress epithelial ER activity, we further asked whether stromal PR expression is downregulated in the *COUP-TFII* conditional mutants. Indeed, PR expression is reduced significantly in the uterine stroma, while no obvious change is seen in the epithelium. This finding indicates an indispensable role for stromal COUP-TFII in the maintenance of progesterone suppression of uterine epithelial ER activity, a prerequisite for the establishment of normal uterine receptivity. This study also substantiates the importance of epithelial–stromal cross-communication and sheds new light on a complex signaling circuit that spans the uterine epithelial–stromal divide that is indispensable for the development of the receptive uterus and subsequent decidualization. In addition, not only does this finding further our understanding of steroid hormonal control of uterine receptivity, but it provides a novel signaling paradigm for steroid hormonal dysregulation shown to underlie such female reproductive pathologies as endometriosis and endometrial, ovarian, and breast cancers.

## Results

### COUP-TFII Expression Is Efficiently Ablated in the Uterus of *COUP-TFII* Mutant Mice

**Figure 1:**
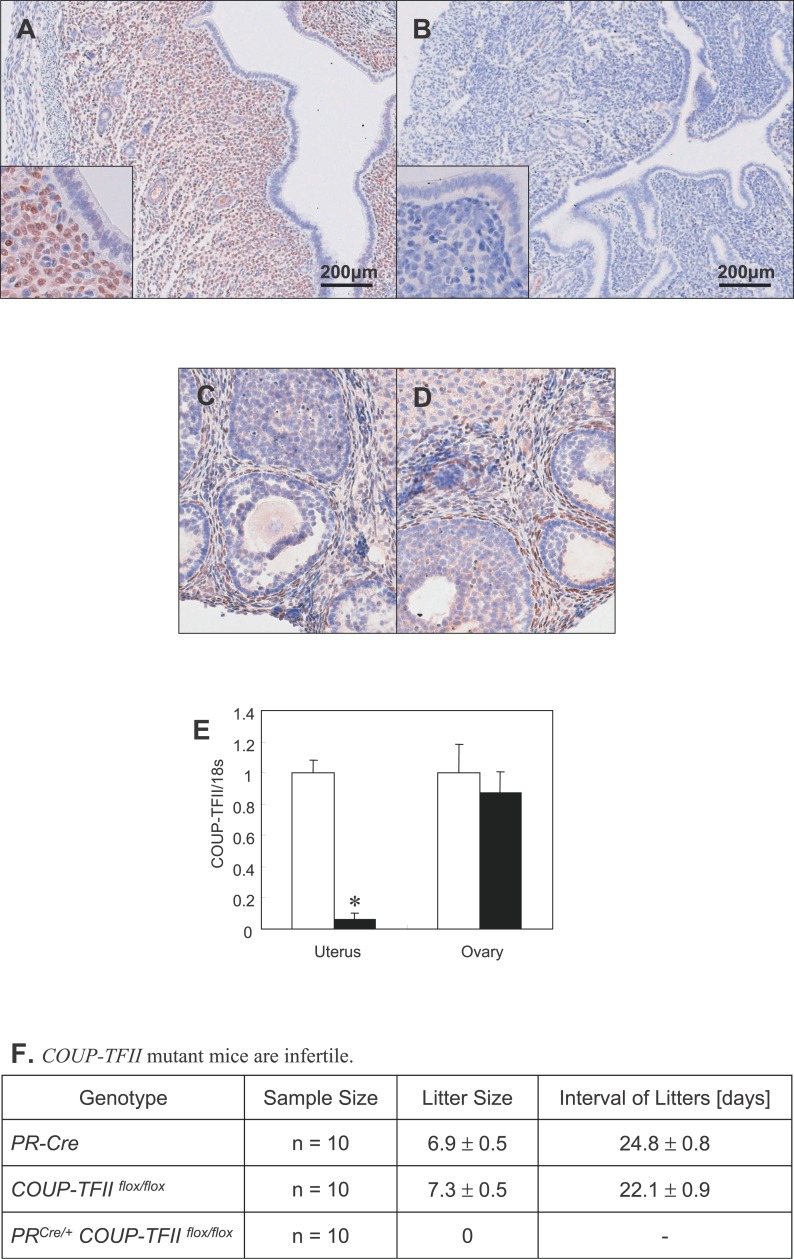
COUP-TFII Is Efficiently Deleted by PR-Cre, and Resulting Mutants Are Infertile However, COUP-TFII is not deleted in the ovary and, thus, mutants exhibit no ovarian defects. (A–B) Immunohistological detection of COUP-TFII in the uterus. (A) Control, 4.5 dpc. COUP-TFII is highly expressed in the endometrial stroma, but is undetectable in the epithelial compartment. (B) Mutant, 4.5 dpc. COUP-TFII expression is efficiently ablated in the mutant uterus. (C–D) Immunohistological detection of COUP-TFII in the ovary. (C) Control, 4.5dpc. COUP-TFII is highly expressed in the theca cell layer. (D) Mutant, 4.5dpc. COUP-TFII expression is maintained in the theca cell layer. (E) The gene expression of *COUP-TFII,* assayed by quantitative real-time RT-PCR. *COUP-TFII* expression is efficiently ablated in the uterus but not affected in the ovary. White bar: control; black bar: mutant. *p < 0.001 (*t*-test, *n* = 9). (F) Summary of breeding studies. *PR^Cre/+^ COUP-TFII^flox/flox^* mutants are infertile, while both controls are normal in fertility.

Conditional knockout mice of *COUP-TFII*, *PR^Cre/+^ COUP-TFII^flox/flox^* were generated by crossing *PR-Cre* knockin mice with *COUP-TFII^flox/flox^* mice. PR is highly expressed in the uterine stroma and epithelium, while COUP-TFII is highly expressed in the stroma, but rarely expressed, if ever, in the uterine epithelium [18,22]. Immunohistochemistry of the reproductive tract indicated that *COUP-TFII* is efficiently ablated in the stroma of the mutant uterus by the PR-Cre (Figure 1A and 1B). It is also evident from this figure that COUP-TFII is highly expressed in the stroma compartment but is hardly detectable in the luminal and the glandular epithelia of the uterus. In contrast, PR is expressed in the granulosa cells, while COUP-TFII is expressed in the theca cells of the ovary [18,22]. Since PR and COUP-TFII are not expressed in the same cell, *COUP-TFII* is not ablated in the theca cells. As expected, the expression of COUP-TFII in the theca cells of the ovary is not altered in the conditional *COUP-TFII* mutants comparison with controls as shown by immunostaining (Figure 1C–1E). To ensure there is no disruption of ovarian function in *COUP-TFII* mutants, we transferred ovaries from control and mutant mice to wild-type recipients and observed reproduction for a 6-mo period. Healthy newborns were yielded from *PR^Cre/+^ COUP-TFII^flox/flox^* ovaries in a similar manner as the controlled *PR^Cre/+^* and *PR^Cre/+^ COUP-TFII^flox/+^* ovaries (Table 1). In addition, the litter size from the mutant ovaries was not significantly reduced compared with that of controls, indicating that the *PR^Cre/+^ COUP-TFII^flox/flox^* females have no ovarian defects (Table 1). Thus, the implantation failure observed in our conditional mutants is likely due to impaired uterine, but not ovarian, function.

**Table 1:**
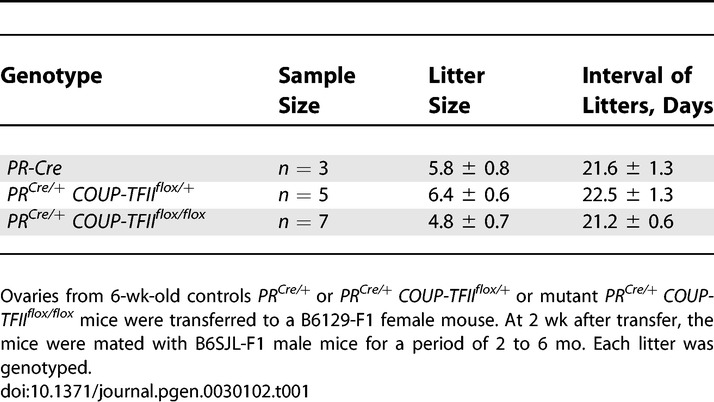
Ovaries of *COUP-TFII* Mutant Mice Were Functionally Normal Compared with Control Mice

### *COUP-TFII* Mutants Are Infertile Due to Failure of Embryo Attachment

*PR^Cre/+^ COUP-TFII^flox/flox^* mutant mice and *COUP-TFII^flox/flox^* control mice were mated with wild-type males (B6SJLF1; Taconic) and observed for 6 mo to compare breeding capacity. *PR-Cre* mice were also used as a control to distinguish the contribution of the *PR-Cre* allele. Pups were not born from mutant females, while both types of controls gave birth regularly (Figure 1F), indicating that ablation of *COUP-TFII* in the uterus leads to infertility. The hormone profile during pregnancy showed no significant difference in estradiol (control, 45.3 ± 3.8 pg/ml; mutant, 47.9 ± 3.2 pg/ml; *n* = 14, 3.5 d postcoitus [3.5 dpc]) and progesterone levels (control, 15.8 ± 4.4 ng/ml; mutant, 18.6 ± 2.9 pg/ml; *n* = 12, 3.5 dpc) between mutants and controls, further supporting the fact that *PR^Cre/+^ COUP-TFII^flox/flox^* mice have no obvious ovarian defect as stated above (Figure 1C–1E; Table 1).

**Figure 2:**
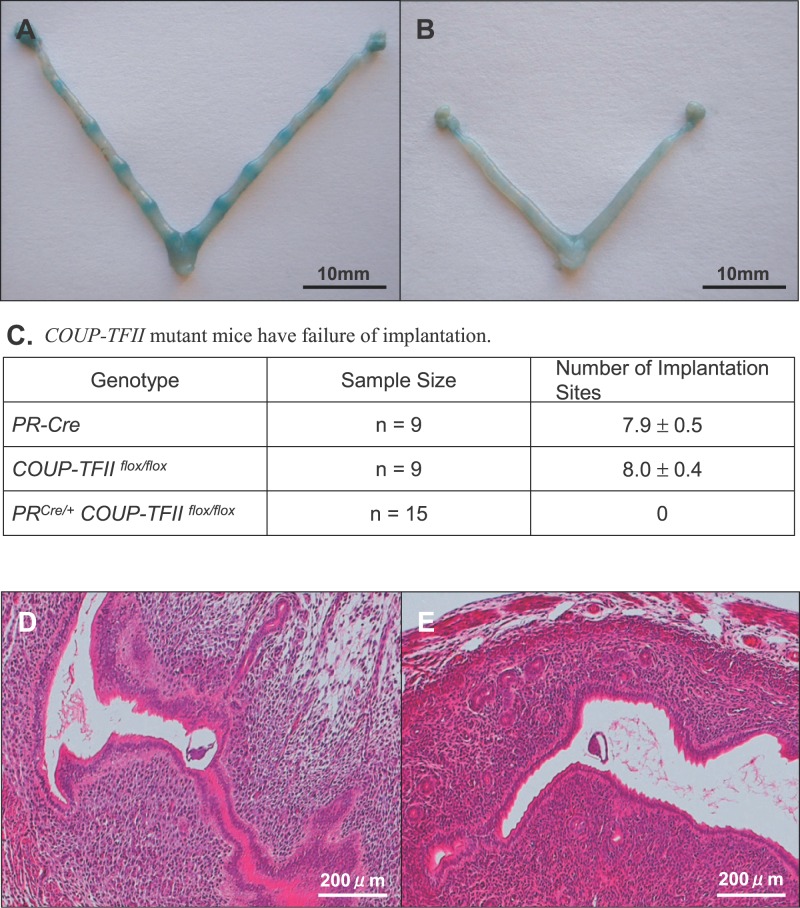
COUP-TFII Mutants Are Defective in Embryo Attachment *PR^Cre/+^ COUP-TFII^flox/flox^* mutants have implantation failure. (A) Control, 4.5 dpc. Implantation sites are visualized as blue spots. (B) Mutant, 4.5 dpc. Mutant uterine horns have no implantation sites and appear small in size. (C) Summary of implantation study. (D) Control, hematoxylin and eosin staining (HE), 4.5 dpc. Embryo is attached to the uterine lumen and the surrounding stroma is permeated as an initial decidual response. (E) Mutant, HE, 4.5 dpc. No embryos are attached to the mutant uterine lumen. A total of 20 embryos were examined in each group.

To dissect the cause of infertility, we examined whether embryos properly attach to the uterine lumen, an early event of pregnancy that is initiated at midnight of pregnancy day 4 (4 dpc). We dissected mice on the morning of pregnancy day 5 (4.5 dpc) and counted the number of implantation sites by injecting Chicago Blue dye. Implantation sites were not detected in the mutant uterine horns, while normal implantation sites were scored in the controls (Figure 2A–2C). Histological examination also showed embryos failed to attach to the uterine lumen of mutant mice, while normal attachment and induction of the decidual response was observed in all controls (Figure 2D–2E). Embryo-attachment failure is most likely caused by an altered uterine receptivity response in the mutant model, since the blastocyst still contains an unaltered *COUP-TFII* allele, and even mutant embryos are able to implant in wild-type mothers as previously described [17].

### *COUP-TFII* Mutants Lose the Potential for Decidualization

**Figure 3:**
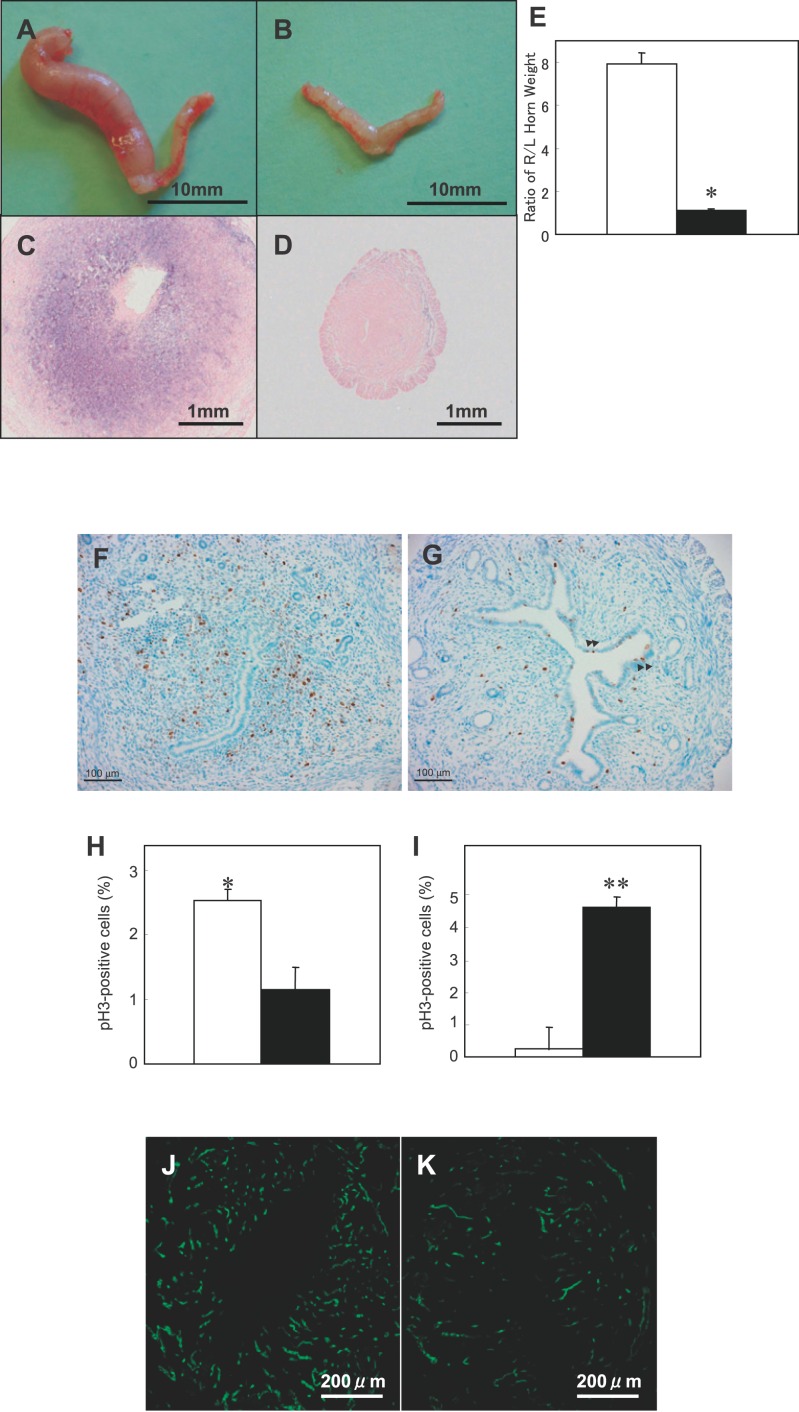
COUP-TFII Mutants Are Defective in Decidualization (A–E) *PR^Cre/+^ COUP-TFII^flox/flox^* mutants have decidualization failure. (A) Control, 48 h after stimuli. The right horn (R) was stimulated, and the left horn (L) was unstimulated. Only right horn is decidualized. (B) Mutant, 48 h after stimuli. Neither right (stimulated) nor left (unstimulated) horn is decidualized. (C) Control, right horn. Alkaline phosphatase activity is detected in the decidualized stroma. (D) Mutant, right horn. No alkaline phosphatase activity is detected in the mutant uterus. (E) Ratio of right to left horn in weight. Mutant uterine horns fail to decidualize. White bar: control; black bar: mutant. *p < 0.001 (*t*-test, *n* = 6). k–o, cell proliferation is altered in *PR^Cre/+^ COUP-TFII^flox/flox^* mutants. (F) Control, phospho-H3, 3.5 dpc. (G) Mutant, phospho-H3, 3.5 dpc. Some positive cells in the epithelium of the mutant are marked by an arrowhead. (H–I) Percentage of phospho-H3–positive cells in the stroma (H) and in the epithelia (I). The number of cells were counted on multiple sections and averaged in each mouse. Stromal cell proliferation is decreased, while epithelial proliferation is enhanced in the mutant uterus. White bar: control; black bar: mutant. *p = 0.007; **p = 0.001 (*t*-test, *n* = 7). (J–K) *PR^Cre/+^ COUP-TFII^flox/flox^* mutants have an angiogenesis defect. (J) Control, 3.5 dpc. Lectin was intravenously administered and adhered to the inner lumen of vessels. (K) Mutant, 3.5 dpc. Vascular density is apparently reduced in the mutant uterus.

Decidualization is the subsequent step in the implantation process [23]. Although it is not possible to compare the decidual response in natural pregnancies of these mice, decidualization was assayed after hormonal induction [24]. Induction of decidualization was normal in *COUP-TFII^flox/flox^* control mice using two types of stimuli (oil injection into the uterine lumen or needle scratching on the antimesometrial side of luminal epithelia). Uterine horns of *PR^Cre/+^ COUP-TFII^flox/flox^* mutant mice failed to decidualize under treatment of either stimuli (Figure 3A, 3B, and 3E), and alkaline phosphatase activity, an indicator of stromal cell differentiation in response to decidualization, was absent (Figure 3C and 3E). In addition to the failure of decidualization, stromal cell proliferation is also affected since the size of mutant uterine horns appear small at 4.5 dpc (Figure 2A and 2B). Immunostaining of phosphorylated histone H3 (phospho-H3) demonstrated that stromal cell proliferation was significantly decreased as indicated by the phospho-H3–positive cells (Figure 3F and 3G). The numbers of phospho-H3–positive cells in stroma are quantified and shown in Figure 3H. In contrast to the stroma, the numbers of phospho-H3–positive cells in the epithelia are increased in the mutant (Figure 3F and 3G). The increase in the numbers of proliferating cells in the mutant epithelium are quantified and shown in Figure 3I. In addition to the decreased proliferation in the stroma, vessel density visualized by lectin staining was also lower in the mutant uterus (Figure 3J and 3K). Reduced angiogenesis could partly contribute to the decrease in size of the uterine horn.

### BMP2 Is the Major Downstream Effector of COUP-TFII for Decidualization

**Figure 4:**
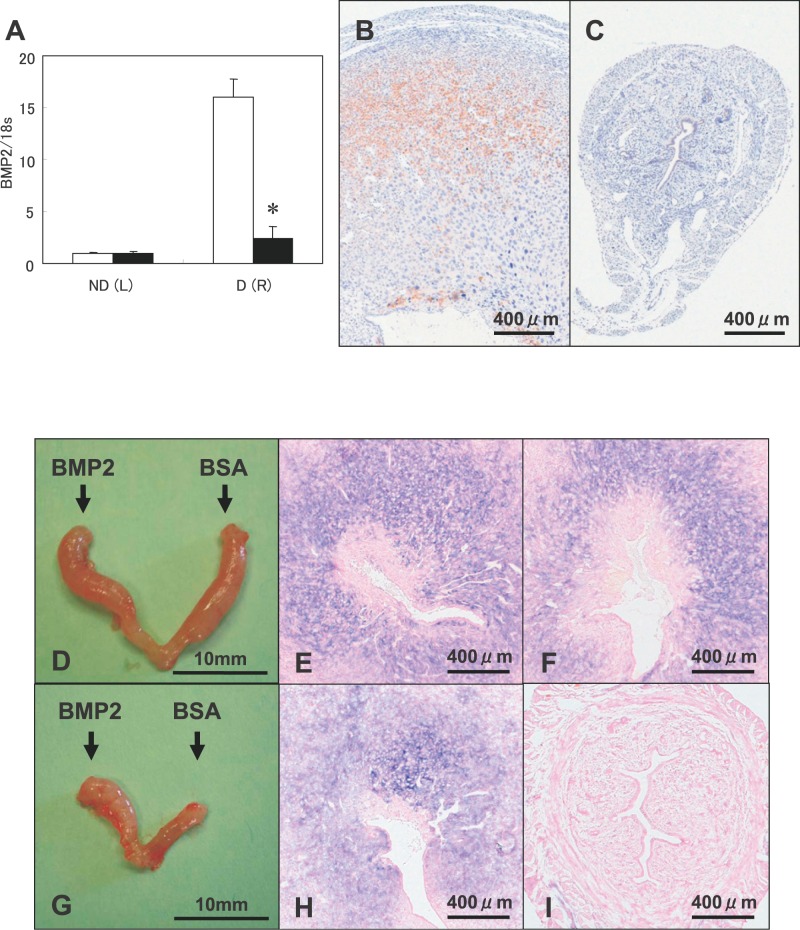
BMP2 Mediates COUP-TFII in Decidualization (A–C) BMP2 induction is diminished in the mutant uterus. (A) The gene expression of *Bmp2* in the stimulated horn (D) and the unstimulated horn (ND), assayed by quantitative real-time RT-PCR at 48 h after stimuli. *Bmp2* expression is induced by stimuli for decidualization in the control, but is not sufficiently induced in the mutant uterus. White bar: control; black bar: mutant. *p < 0.001, (*t*-test, *n* = 6). (B) Control, right horn, 72 h after stimuli. BMP2 is immunohistologically detected in the secondary decidual zone. (C) Mutant, right horn, 72 h after stimuli. BMP2 is not detected in the mutant stroma. (D–I) Decidualization is rescued by the administration of recombinant human BMP2. (D) Control, 48 h after stimuli. The right horn was treated with BMP2, and the left horn was treated with BSA (vehicle). Both horns are decidualized. (E) Control, right horn. (F) Control, left horn. Alkaline phosphatase activity is detected in both uterine horns. (G) Mutant, 48 h after stimuli. Only the right horn is decidualized. (H) Mutant, right horn. (I) Mutant, left horn. Alkaline phosphatase activity is detectable in the BMP2-treated horn, but not in the BSA-treated horn of mutant uterus.

BMP2 is a known specific marker for decidualization in the uterus, and its expression is greatly induced upon decidualization [25,26]. To explore the molecular mechanism of decidualization failure in the *COUP-TFII* mutant mice, we asked whether expression of BMP2 is altered. Basal *Bmp2* expression levels were unaffected in the mutants in comparison with the controls. However, the induced expression of *Bmp2* upon decidualization was greatly diminished in the mutant uterus (Figure 4A). Immunohistochemistry confirmed no stromal expression of BMP2 in the mutant uterus (Figure 4B and 4C). The above results suggest that BMP2 is a downstream target of COUP-TFII that regulates the decidual response. To address this, we asked whether BMP2 could rescue the decidualization defect exhibited by the *COUP-TFII* conditional mutant. Along with artificially stimulating the uterus, recombinant human BMP2 was administered into the uterine lumen. Mice were dissected 48 h later, and the decidual response was measured. BMP2 treatment restored the decidual response in the mutant uterine horns (Figure 4D and 4G) as measured by the enhancement of alkaline phosphatase activity in the stimulated horns, while no activity was detected in the vehicle (BSA)–treated mutant horns (Figure 4E, 4F, 4H, and 4I). These results strongly support that BMP2 is a major COUP-TFII effector that lies downstream of COUP-TFII to mediate uterine decidualization. BMP2 has also been shown as a downstream target of hedgehog signaling in other tissues [27,28], and conditional ablation of *Bmp2* results in decidualization defects, but embryo attachment is unaffected (Lee et al., unpublished data). Therein, our finding provides new evidence in support of the existence of a uterine Ihh–COUP-TFII–BMP2 axis that is required for decidualization.

### Estrogen Activity Is Enhanced in the Uterine Epithelia of *COUP-TFII* Mutants

**Figure 5:**
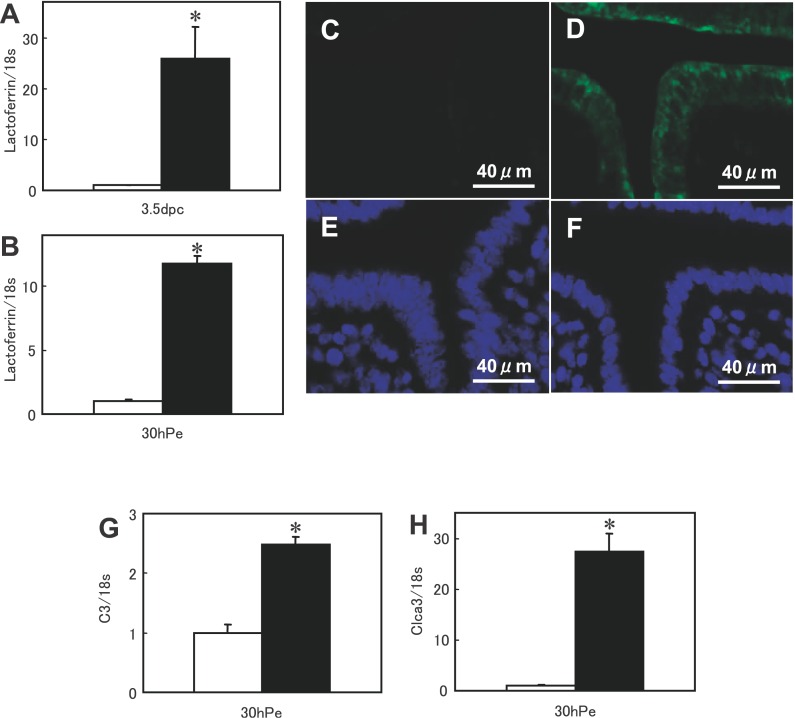
COUP-TFII Regulates ER Activity in the Epithelia Estrogen-responsive genes are upregulated in the mutant uterine epithelia. (A) The gene expression of *Ltf,* assayed by qRT-PCR at 3.5 dpc. *Ltf* expression is high in the mutant uterus. White bar: control; black bar: mutant. *p = 0.012 (*t*-test, *n* = 6). (B) *Ltf,* qRT-PCR, 30 hPe. *Ltf* expression is consistently high in the mutant uterus. White bar: control; black bar: mutant. *p < 0.001 (*t*-test, *n* = 9). (C) Control, immunohistological detection of lactoferrin, 30 hPe. (D) Mutant, lactoferrin. (E) Control, DAPI. (F) Mutant, DAPI. Upregulated expression of lactoferrin is observed in the epithelial compartment. (G) The gene expression of *C3,* assayed by qRT-PCR at 30 hPe. *C3* expression is high in the mutant uterus. White bar: control; black bar: mutant. *p = 0.015 (*t*-test, *n* = 9). (H) *Clca3,* qRT-PCR, 30 hPe. *Clca3* expression is high in the mutant uterus. White bar: control; black bar: mutant. *p = 0.002 (*t*-test, *n* = 9).

The lack of embryo attachment indicates that ablation of *COUP-TFII* not only affects the physiology of uterine stromal cells but also affects the endometrial epithelial compartment. One of the major roles of progesterone is to down-regulate ER activity in the uterine luminal epithelium, which consequently opens the uterine receptivity window. Since *COUP-TFII* mutants have a receptivity defect (Figure 2A–2E), we wondered whether COUP-TFII is a mediator of progesterone's suppression of ER activity in the epithelia. If so, ER activity in this compartment should increase in *COUP-TFII* mutants. To address this, the expression level of estrogen-responsive genes was examined by quantitative real-time RT-PCR analysis (qRT-PCR). The expression of *lactoferrin (Ltf),* a known estrogen-responsive target in the uterine epithelia [29], is significantly elevated in the mutant uterus at 3.5d pc (Figure 5A). To exclude the possible involvement of other factors, we also examined the expression of *Ltf* in mice exogenously treated with hormones, mimicking 3.5 dpc of pregnancy (30 h after progesterone and estrogen [Pe] treatment; see Materials and Methods). Although the fold changes vary, *Ltf* expression level is consistently significantly higher in mutant mice (Figure 5B). Immunohistological staining detected high lactoferrin expression in mutant epithelia (Figure 5C–5F), demonstrating that estrogen activity is indeed enhanced in the uterine epithelial compartment. Other well-documented estrogen-responsive genes in the uterine epithelia, including *complement component 3 (C3)* and *chloride channel calcium activated 3 (Clca3)* [30,31], were also elevated in the mutant mice (Figure 5G and 5H), indicating that estrogen activity is upregulated in the uterine luminal epithelium of mutant mice.

### High Estrogen Activity Alters Uterine Receptivity in *COUP-TFII* Mutants

**Figure 6:**
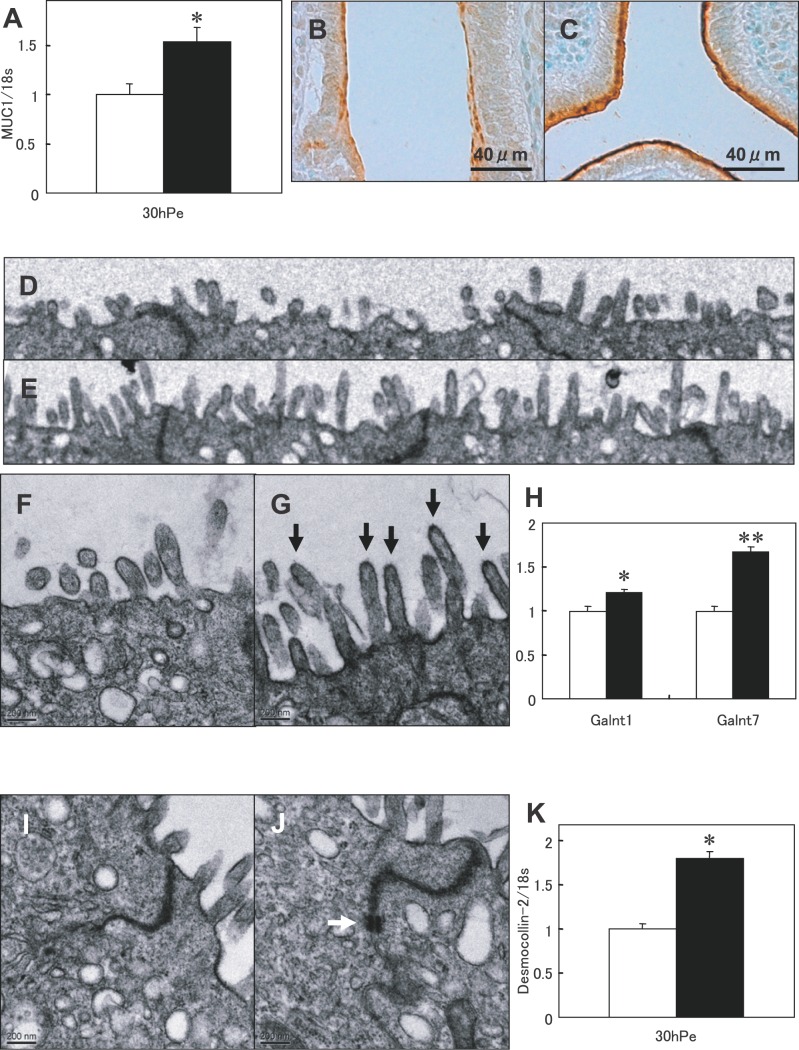
Uterine Receptivity Is Disrupted by High Estrogen Activity in the Mutant Uterine Epithelia (A) The gene expression of *Muc1,* assayed by qRT-PCR at 30 hPe. *Muc1* expression is high in the mutant uterus. White bar: control; black bar: mutant. *p = 0.041 (*t*-test, *n* = 9). (B) Control, immunohistological detection of MUC1, 30 hPe. (C) Mutant, MUC1, 30 hPe. MUC1 is detected in the apical surface of uterine epithelia, and its expression level is high in the mutants. (D) Control, EM, 30 hPe. Membrane flattening is observed in the surface of uterine epithelia. (E) Mutant, EM, 30 hPe. Mutant uterine epithelia fail to undergo membrane flattening. (F) Control, EM, 30 hPe. (G) Mutant, EM, 30 hPe. Mutant microvilli are excessively coated with glycocalyx (arrow). (H) The gene expression of *Galnt1* and *Galnt7,* assayed by qRT-PCR at 30 hPe. The expression of both enzymes is high in the mutant uterus. White bar: control; black bar: mutant. **p = 0.008, **p < 0.001 (*t*-test, *n* = 9). (I) Control, EM, 30 hPe. (J) Mutant, EM, 30 hPe. Mutant uterine epithelia exhibit the persistent presence of desmosome (arrow). (K) The gene expression of *Dsc2*, assayed by qRT-PCR at 30 hPe. *Dsc2* expression is high in the mutant uterus. White bar: control; black bar: mutant. *p = 0.001 (*t*-test, *n* = 9).

Mucin 1 (MUC1) is known to be one of the important markers determining uterine receptivity [32]. MUC1 is an estrogen-responsive target, and its expression is attenuated at the time of implantation to facilitate epithelial remodeling [33,34]. Persistent expression of MUC1 during the peri-implantation period prevents uterine receptivity and embryo attachment [32]. qRT-PCR showed high expression levels of *Muc1* in the mutant uterus (Figure 6A). In addition, immunohistochemistry detected high expression levels of MUC1 in the apical surface of mutant luminal epithelia (Figure 6B and 6C). These results suggest that high estrogen activity might be the underlying cause of the uterine receptivity defect displayed by the mutant model. Consistent with this notion, *Clca3,* a gene important for the overproduction of mucus protein [35], is also shown to be highly upregulated in the mutant uterus (Figure 5H). Therefore, upregulation of many ER target genes suggests that stromal COUP-TFII is essential for the PR-mediated downregulation of ER activity in the epithelium to open up the receptivity window.

The membrane transformation of uterine epithelia is well documented as a marker of uterine receptivity [36]. Long microvilli of the epithelial surface are characteristically present under estrogen influence, while progesterone shortens these structures. Microvilli flattening occurs before implantation and is an important process to facilitate embryo attachment [36]. Electron microscope (EM) studies revealed that mutant epithelia fail to undergo appropriate remodeling to flatten the microvilli (Figure 6D and 6E). In addition, mutant microvilli exhibit increased glycocalyx expression (Figure 6F and 6G), which is consistent with high expression of MUC1 [36]. Both MUC1 expression and glycocalyx formation prevent embryo attachment [34,37,38]. It has been reported that a series of glycosylation enzymes are involved in the glycosylation of mucins, and among them, UDP-*N*-acetyl-alpha-*D*-galactosamine: polypeptide *N*-acetylgalactosaminyltransferase 1 (GALNT1) is a key enzyme [39]. We examined *Galnt1* expression in qRT-PCR and observed that its expression level is increased by 20% (Figure 6H), and, most remarkably, another glycosylation enzyme *Galnt7* expression is upregulated by almost 70% in the mutant uterus (Figure 6H). Although it has not been well established in the mouse uterus, activation of GALNT7 catalytic activity requires prior glycosylation by other enzymes [40], and GALNT7 cooperatively functions with GALNT1 [41]. The high expression of these enzymes might account for hyperglycosylation of the apical surface of the mutant uterine luminal epithelium. Another important parameter for uterine epithelial maturation is the presence of desmosomes [36,42,43], adherent junctions of the lateral plasma membrane. Desmosomes are normally lost before implantation to facilitate embryo invasion into the uterine stroma. However, desmosomes are persistently present in the mutant epithelia (Figure 6I and 6J). As expected, the expression level of *desmocollin-2 (Dsc2),* one of the ubiquitous desmosomal components [44], is high in the mutant uterus (Figure 6K). This inappropriate regulation of *Dsc2* might contribute to desmosome dysregulation. Taken together, the high estrogen activity observed in the mutant epithelium alters the uterine receptivity in the mutant mice, which is reflected by striking structural abnormalities in the apical–lateral regions of mutant luminal epithelial cell.

### Downregulation of PR Expression in the Uterine Stroma of *COUP-TFII* Mutants

**Figure 7:**
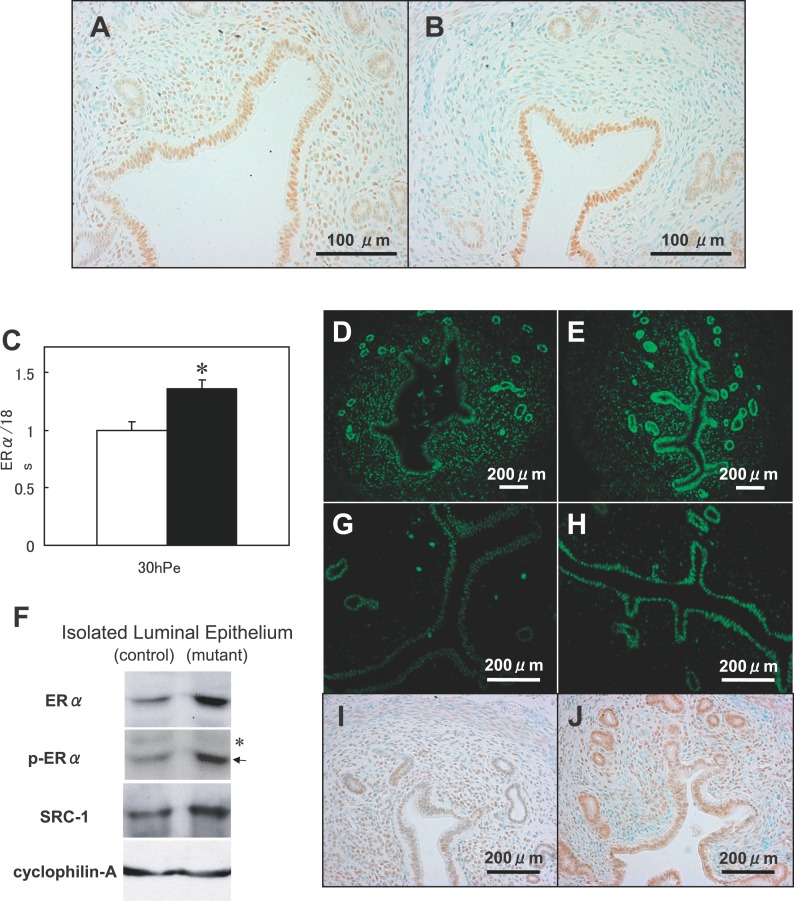
COUP-TFII Regulates the Expression of ERα and SRC-1 in the Uterine Epithelia (A–B) Immunohistological detection of PR at 30 hPe. (A) Control (*PR^Cre/+^ COUP-TFII^+/+^*) (B) Mutant (*PR^Cre/+^ COUP-TFII^flox/flox^*). The expression of PR is significantly reduced in the stroma of *COUP-TFII* mutant uterus. The same result is observed under another comparison between *COUP-TFII^flox/flox^* and *PR^Cre/+^ COUP-TFII^flox/flox^*). (C) The gene expression of *Esr1*, assayed by qRT-PCR at 30 hPe. *ERα* expression is increased in the whole mutant uterus. White bar: control; black bar: mutant. *p = 0.012 (*t*-test, *n* = 9). (D) Immunohistological detection of ERα at 30 hPe of control uterus. (E) ERα expression is increased in the mutant uterus. (F) Western blot analysis of ERα, phospho-ERα, and SRC-1 in isolated luminal epithelial cells. Intensity of signals was measured by National Institutes of Health image software (http://rsb.info.nih.gov/nih-image) and normalized by cyclophilin-A. ERα, phospho-ERα, and SRC-1 were increased 2.5-fold, 2.4-fold, and 1.6-fold in the isolated luminal epithelia (LE) of the mutant uterus, respectively. *Nonspecific band. Arrow indicates the pERα, which is confirmed by reprobing with anti-ERα antibody. (G) Immunohistological detection of phospho-ERα at 30 hPe of control uterus. (H) Level of pERα is increased in the mutant uterine epithelia. (I) Immunohistological detection of SRC-1 at 30 hPe of control uterus. (J) Increased SRC-1 is detected in the epithelial compartment of mutant uterus.

PR in the stroma has been implicated to play a critical role in modulating ER activity in the epithelium [6,7]. Since the activity of ER is enhanced in *COUP-TFII* mutants, an important question is whether ablation of *COUP-TFII* in the uterine stroma alters the expression level of stromal PR. To address this possibility, we used PR-specific immunostaining to assess the expression of PR in the uterus of controls and mutants. The result clearly shows that the expression level of PR is significantly reduced in the stroma of *COUP-TFII* mutants (Figure 7A and 7B). In contrast, there is no significant change in the PR expression levels in the luminal epithelium or the glandular epithelium. This result indicates that downregulation of PR in the stroma in the absence of COUP-TFII could disrupt stromal–epithelial interactions and contribute to the enhanced ER activity.

### The Expression of Epithelial ER and Its Coactivator SRC-1 Is Upregulated in *COUP-TFII* Mutants

In an attempt to further dissect the molecular mechanism of enhanced estrogen activity in the mutant uterine epithelium, we first asked whether uterine ERα levels are altered in mutants. qRT-PCR showed a 40% increase in levels of *ERα (Esr1)* mRNA in the whole-uterine tissues of *COUP-TFII* mutants (Figure 7C). Immunohistological staining using ERα-specific antibody further confirmed an increased expression of ERα in the epithelial compartment of *COUP-TFII* mutants (Figure 7D and 7E). To quantify the difference in expression levels, we isolated uterine epithelia from whole uterus and examined ERα expression by western blot analysis. The result showed that ERα expression is increased 2- to 3-fold in the mutant uterine epithelia (Figure 7F). To further ask whether these receptors are activated or not, we examined the phosphorylation status of ERα using antiphosphorylated ERα antibody and observed increased phosphorylation of ER in the uterine epithelium of *COUP-TFII* mutants (Figure 7F–7H). Increased phosphorylation levels of ER seem proportional to increased expression levels of ER, but this modification has been shown to couple with growth factor signaling, which might be controlled under paracrine mechanism [45] and is less likely to be an autophosphorylation; therefore, this finding really supports the notion that stromal–epithelial communication is dysregulated in the *COUP-TFII* mutant uterus. In addition to ER, members of the steroid receptor coactivator (SRC)/p160 family, SRC-1 and SRC-2, have been shown to play a major role in regulating ER activity and uterine function [46–48]. Thus, we examined the expression of coactivators by both immunohistochemistry and western blot analysis. We showed that SRC-1 is upregulated in mutant uterine epithelia (Figure 7F, 7I, and 7J), while SRC-2 and SRC-3 are unchanged (unpublished data). Taken together, the increase in ER, phosphorylated ER, and SRC-1 levels in the mutant uterine epithelium can together contribute to enhanced uterine ER activity in the *COUP-TFII* mutant.

## Discussion

Uterine receptivity has been intensely studied in recent years because of its clinical importance [49,50]. Mouse models generated by gene-knockout technology revealed that multiple factors are involved in this process [1–3]. Although individual factors have proven to be essential for uterine receptivity, most of them are directly or indirectly controlled by estrogen and/or progesterone. Therefore, we assume that the balance in activities between these two hormones is a major determinant of successful uterine receptivity. Indeed the levels of estrogen used in in vitro fertilization procedures have recently been suggested as a likely contributor to lower pregnancy successes when using artificial reproductive techniques [51]. This reappraisal prompts the question of how to control estrogen activity during the peri-implantation period so that higher success rates with in vitro fertilization can be achieved. An important step toward addressing this question is to define the mechanism by which progesterone modulates estrogen activity in the uterine epithelium. Understanding this pivotal control mechanism would enable the formulation of better clinical protocols to induce and preserve the receptive uterus.

**Figure 8:**
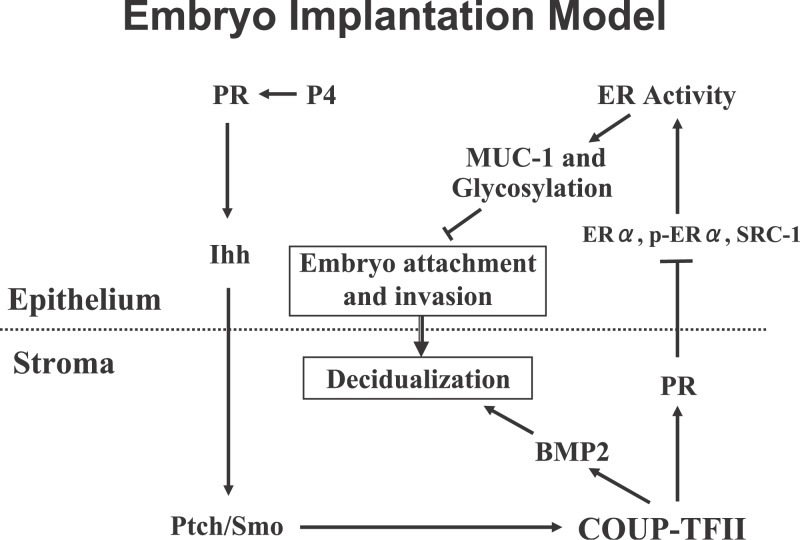
Working Model of COUP-TFII in Mediating Progesterone Function in the Uterus Progesterone activates Ihh–Ptch signaling to induce COUP-TFII expression in the stroma compartment. COUP-TFII, in turn, regulates stromal cell differentiation (decidualization) through the induction of BMP2. COUP-TFII also mediates the suppression of epithelial estrogen activity through inhibiting the expression of SRC-1 and ERα as well as ERα activation, which allows the uterine epithelia to undergo transformation and gain receptivity for embryo attachment.

Based on the findings described herein, we propose a new model to explain estrogen and progesterone control of uterine implantation. In this model, progesterone activates the Ihh–COUP-TFII–BMP signaling axis to elicit stromal cell differentiation that is required for decidualization. Importantly, COUP-TFII also mediates progesterone-induced suppression of epithelial estrogen action through decreasing epithelial ER and SRC-1 levels and inhibition of ER activation (phosphorylation) during the peri-implantation period (Figure 8). All these effects are likely due to its regulation of stromal PR level, which was shown to be responsible for the downregulation of ER activity [6,7]. Because COUP-TFII is expressed in the stroma, a paracrine mechanism of action is proposed by which stromal-derived COUP-TFII controls epithelial ER activity through as-yet-unknown mediator(s) that transmits the inhibitory signal from the stromal to the epithelial compartment. Although beyond the scope of this study, identification of this paracrine signal represents the next most important step to fully understand the complete circuitry of progesterone/estrogen action in reproduction.

The model of progesterone signaling described here is most likely oversimplified. Many other players in both epithelial and stroma compartments may also participate in the overall regulation. For example, ER in the stromal compartment and the very low expression of COUP-TFII, if any, in the epithelial compartment, may also participate in some way in this scheme. Only by compartmental-specific deletion of these genes can we validate and dissect the contributions of these proteins to embryo implantation in the future. As reported previously, COUP-TFII is regulated by progesterone through Ihh signaling, which emanates from the epithelial compartment of the uterus, but we should not underestimate the roles of stromal PR because tissue-recombinant experimental models have demonstrated that epithelial estrogen activities are suppressed by stromal PR [6,7]. Indeed decreased expression of stromal PR is expectedly observed in *COUP-TFII* mutant mice, which was examined under proper comparison (*PR^Cre/+^* versus *PR^Cre/+^ COUP-TFII^flox/flox^*; Figure 7A and 7B), although detailed regulatory mechanism of this interdependence has yet to be defined. In addition, leukemia-inhibiting factor (LIF), which has been well documented, may also participate in this scheme. *Lif*-null mice exhibit defects in embryo attachment, in which the specific ultrastructural and immunohistological features associated with a receptive uterus are lost [52]. Since observed phenotypes in *Lif*-null mice are similar to *COUP-TFII* mutant mice, LIF also could be placed in our scheme. LIF is known to be estrogen responsive; when examined, we did not find significant changes in LIF by qRT-PCR and by immunocytochemistry in mutants in comparison to the controls. It is possible that more complex mechanism underlies our model, but it is still unequivocal that COUP-TFII has access to the principal part of steroid receptor regulation in the uterine biology.

The finding that COUP-TFII antagonizes ER action is intriguing. ER has been shown to regulate the expression of many glycoproteins during the peri-implantation period [34,53]. Downregulation of the expression of such glycoproteins (including MUC1) is known to pave the way for remodeling of the epithelial surface to facilitate embryo attachment. Although COUP-TFII has been shown to compete with ER binding in vitro in the regulation of *Ltf* [54,55], COUP-TFII is not expressed in the same compartment as lactoferrin and MUC1, and thus it is unlikely that it regulates their expression directly in vivo. Using tissue-recombinant studies, Buchanan et al. showed that epithelial lactoferrin expression is not only regulated by epithelial ER but also regulated by stromal ER [56]. This raises the possibility that COUP-TFII might compete with stromal ER and alter the epithelial ER function. Another possible mechanism is that COUP-TFII regulates local estrogen levels, since COUP-TFII has been shown to compete with SF-1 to regulate aromatase expression [57]. However, aromatase expression was not altered in the *COUP-TFII* conditional mutant mice (unpublished data). We also showed that the expression of ER, phosphorylated ER, and SRC-1 are all increased in the *COUP-TFII* mutants. Enhanced expression of these molecules will no doubt contribute to the observed increased ER activity and the subsequent activation of the downstream ER targets. Since COUP-TFII is highly expressed in the stroma but is barely detectable in the epithelia, the up-regulation of ER activity in the epithelium is unlikely a consequence of direct regulation of the above molecules by COUP-TFII. It is more likely that the stromal COUP-TFII regulates PR to control a paracrine signal, which acts through its epithelial receptor to suppress epithelial ER activity as well as ER and its coregulator expression. Unlikely as it might be, we can not exclude the possibility that the low levels of epithelial COUP-TFII expression is sufficient to synergize with other epithelial factors to suppress epithelial ER activity directly.

In conclusion, COUP-TFII controls early molecular and cellular changes in the uterus that are required for embryo implantation and subsequent decidualization. Based on our previous observation that COUP-TFII is a mediator of the Shh pathway in motor neurons and the stomach [11,14], it is not surprising that COUP-TFII mediates progesterone–Ihh signaling to regulate decidualization. We also show that BMP2 can rescue the decidual defect elicited by the loss of COUP-TFII, which places BMP2 downstream of the COUP-TFII pathway. Unexpectedly, stromal COUP-TFII also promotes PR expression to mediate progesterone-induced suppression of estrogen activity in the uterine epithelium; local suppression of estrogen activity is required to establish a receptive uterus. Therefore, progesterone control of epithelial estrogen activity is projected from the stromal compartment via COUP-TFII through a complex epithelial–stromal cross-communication pathway. The abnormal increase in estrogen activity following the removal of COUP-TFII may help our understanding of the molecular events that control uterine receptivity as well as female reproductive health.

## Materials and Methods

### Animals and chemicals.

Generation of *COUP-TFII^flox/flox^* mice and *PR-Cre* knockin mice has been previously described [14,22]. To obtain uterine tissues of pregnant mice, we started mating with wild-type males (B6SJLF1; Taconic, http://www.taconic.com) at 7 wk of age and designated the day of vaginal plug as pregnant day 1. Ovariectomy was performed at 6 wk of age and followed by the hormone regimen as described below. For priming with 1 μg of 17β-estradiol (E2; Sigma-Aldrich, http://www.sigmaaldrich.com) was dissolved in 1 ml sesame oil (Sigma-Aldrich), and 0.1 ml was subcutaneously administered in a single dose for each mouse. For daily treatment of Pe, 10 mg progesterone (Sigma-Aldrich) and 67 ng 17β-estradiol (nidatory estrogen(e)) were dissolved in 1 ml sesame oil, and 0.1 ml was subcutaneously administered in a single dose for each mouse. In the implantation study, 1% Chicago Sky Blue 6B (Sigma-Aldrich) was prepared in 0.9% saline, and 0.1 ml was intravenously injected for each mouse before dissection. For the rescue of decidualization, 25 μg recombinant human BMP2 (Fitzgerald Industries International, http://fitzgerald-fii.com) was reconstituted by 10% BSA, and 10 μl was administered for each uterine horn. All procedures for animal study were approved by the institutional animal care guidelines at Baylor College of Medicine. All assays were repeated at least three times.

### Ovary transfer.

We followed the ovary transfer procedure described previously [58]. Ovaries from 6-wk-old controls, *PR^Cre/+^* or *PR^Cre/+^ COUP-TFII^flox/+^* mice, or mutant *PR^Cre/+^ COUP-TFII^flox/flox^* mice were isolated and then transferred to a B6129-F1 female mouse. At 2 wk after transfer, the mice were mated with B6SJL-F1 male mice for a period of 2 to 6 mo. Each litter was genotyped in order to characterize the origin of the pups. When two litters came from the transferred ovary, the mating was stopped and the experiment was considered a success.

### Induction of decidualization.

The details of this method have been previously described [24]. Briefly, after 2 wk of ovariectomy, we first primed mice with 100 ng of estradiol (E2) for 3 d and then started the daily treatment of 1 mg progesterone and 6.7 ng E2 (Pe) 2 d later. Mechanical stimulation was added 54 h after the first Pe treatment (54 hPe), and mice were dissected 48 h later for decidual response measurement. The same hormone regimen was used for exogenous hormone treatment mimicking 3.5 dpc. Tissues were isolated at 30 hPe.

### Immunohistological staining.

Isolated uterine tissues were fixed in 4% paraformaldehyde (PFA)/PBS, dehydrated through graded ethanol, and processed for paraffin embedding. Primary antibodies used in this study are as follows: mouse monoclonal anti-COUP-TFII (1:1,000; Perseus Proteomics, http://ppmx.com), rabbit polyclonal anti–phospho-H3 (1:200; Upstate Biotechnologies, http://www.upstate.com), goat polyclonal anti-BMP2 (1:100; Santa Cruz Biotechnology, http://www.scbt.com), rabbit polyclonal anti-lactoferrin (1:5,000; Abcam, http://www.abcam.com), rabbit polyclonal anti-MUC1 (1:400; Abcam), rabbit polyclonal anti-PR (1:200; Dako, http://www.dako.com), rabbit polyclonal anti-ERα (1:500; Santa Cruz Biotechnology), rabbit polyclonal anti–phosphorylated ERα (S118, 1:100; Abcam), and rabbit polyclonal anti–SRC-1 (1:500; Santa Cruz Biotechnology). Biotinylated antibodies (1:400; Jackson ImmunoResearch, http://www.jacksonimmuno.com) were used as secondary antibodies, followed by horseradish peroxidase–conjugated streptavidin (1:200; Molecular Probes, http://probes.invitrogen.com), and signals were developed with 3,3′-diaminobenzidine (DAB) substrate kit (Vector Laboratories, http://www.vectorlabs.com) or Alexa fluor 488–conjugated tyramide signal amplification (TSA) kit (Molecular Probes). Hematoxylin or methyl green (Vector Laboratories) was used for counterstaining in immunohistochemistry.

### Alkaline phosphatase staining.

Isolated tissues were fixed in 2% PFA/PBS, cryoprotected by 30% sucrose/PBS, and embedded in OCT compounds (Sakura, http://www.sakura.com). The sections were stained with 5-bromo-4-chloro-3-indolyl phosphate (BCIP) and Nitro blue tetrazolium chloride (NBT) solution (pH 9.5; Roche, http://www.roche.com). Nuclear Fast Red (Vector Laboratories) was used for counterstaining.

### Lectin staining.

A total of 25 μg biotinylated-lycopersicon esculentum lectin (Vector Laboratories) was intravenously injected, and then uterine tissues were isolated, fixed in 4% PFA/PBS, cryoprotected by 30% sucrose/PBS, and embedded in OCT compounds. The sections were incubated in horseradish peroxidase–conjugated streptavidin, and signals were developed with the TSA kit.

### Quantitative real-time RT-PCR.

Isolated tissues were quickly stabilized in RNAlater RNA stabilization reagent (QIAGEN, http://www.qiagen.com). Total RNA was extracted using an RNeasy Mini kit (QIAGEN) and reverse transcribed using TaqMan Reverse Transcription Reagents (Applied Biosystems, http://www.appliedbiosystems.com). Gene expression assay was performed by running the ABI PRISM 7700 Sequence Derector System (Applied Biosystems). TaqMan Universal Master Mix reagents and inventoried primer/probe mixture (Applied Biosytems) were used for the reaction. The primers/probes used in this study are the following: *Bmp2* (Mm01962382_s1), *Ltf* (Mm00434787_m1), *C3* (Mm00437858_m1), *Clca3* (Mm00489959_m1), *Muc1* (Mm00449604_m1), *Galnt1* (Mm00489148_m1), *Galnt7* (Mm00519998_m1), *Dsc2* (Mm00516355_m1), *Esr1* (Mm00433149_m1), *COUP-TFII* (Mm00772789_m1). Standard curves were generated by serial dilution of a preparation of total RNA, and mRNA quantities were normalized against 18S RNA determined by using eukaryotic 18S rRNA endogenous control reagents (Applied Biosystems).

### Electron microscopy.

Mice were perfused with 2.5% glutaraldehyde in 0.1 M cacodylate buffer before isolation of uterine tissues. Tissues were cut into 1-mm^3^ pieces, immersed in 2.5% glutaraldehyde and 2.0% formaldehyde in cacodylate buffer with 2 mM CaCl_2_, washed, and then postfixed by 1% OsO_4_ in 0.1 M cacodylate buffer. Next, the tissues were dehydrated through graded ethanol then dehydrated further in propylene oxide, and embedded in Spurr resin. Ultrathin sections (80 nm) were cut with an MT6000 XL Ultramicrotome (RMC Inc., http://www.rmcproducts.com), stained with aqueous uranyl acetate and lead citrate, and examined under a Hitachi-H7500 TEM (http://www.hitachi-hta.com) at 80 kV.

### Isolation of the uterine epithelium.

Uterine luminal epithelial cells were isolated as previously described [59]. Briefly, isolated uteri were placed into Hanks balanced salt solution (HBSS; Ca^2+^-free, Mg^2+^-free), and cut into 1-mm segments. The cut uteri were placed into 1% trypsin/HBSS solution for 1.5 h at 4 °C, and then washed with cold HBSS. The uteri were placed into 20% FBS/HBSS solution for 5 min, and washed with cold HBSS. The uteri were incubated with DNase solution for a minute to break down DNA. The uterine luminal epithelium was gently removed from the uterine stroma under a dissecting microscope.

### Western blot analysis.

The isolated uterine luminal epithelium was lysed with 1× RIPA buffer (150 mM NaCl, 10 mM Tris-Cl [pH 7.5], 0.1% SDS, 1% Triton X-100, 1% deoxycholate, and 5 mM EDTA) containing proteinase inhibitors and phosphatase inhibitors. The whole-uterine luminal epithelial cell lysates were separated on 8% and 15% SDS–polyacrylamide gels and transferred to a nitrocellulose membrane (Amersham Biosciences, http://www.amersham.com). The membranes were blocked in TBST buffer (20 mM Tris [pH 7.6], 137 mM NaCl, and 0.05% Tween 20) containing 1% casein for 1 h and then incubated overnight at 4 °C in 0.5% casein containing primary antibody. The membrane was washed several times with TBST buffer and incubated with horseradish peroxidase–conjugated secondary antibody. After 1 h, the blot was washed several times with TBST buffer and developed with ECL reagents (Amersham Biosciences).

### Steroid hormone assay.

The serum progesterone and estradiol levels were measured with radioimmunoassay by the core laboratory of University of Virginia Center for Research in Reproduction Ligand Assay and Analysis Core.

## Abbreviations

COUP-TFII: chicken ovalbumin upstream promoter transcription factor II
*Dsc2*: *desmocollin-2*
EM: electron microscope
ER: estrogen receptor
*Ihh*: *Indian hedgehog*
LIF: leukemia-inhibiting factor
*Ltf*: *lactoferrin*
*MUC1*: mucin 1
phospho-H3: phosphorylated histone H3
Pe: progesterone and estrogen
PR: progesterone receptor
Ptch: Patched
Shh: sonic hedgehog
SRC: steroid receptor coactivator
