# Correction: COUP-TFII Mediates Progesterone Regulation of Uterine Implantation by Controlling ER Activity

**DOI:** 10.1371/journal.pgen.0030149

**Published:** 2007-08-31

**Authors:** Isao Kurihara, Dong-Kee Lee, Fabrice G Petit, Jaewook Jeong, Kevin Lee, John P Lydon, Francesco J DeMayo, Ming-Jer Tsai, Sophia Y Tsai

Correction for:

Kurihara I, Lee DK, Petit FG, Jeong J, Lee K, et al. (2007) COUP-TFII Mediates Progesterone Regulation of Uterine Implantation by Controlling ER Activity. PLoS Genet 3(6): e102. doi:10.1371/journal.pgen.0030102

The funding statement was incomplete. The full funding statement follows.

**Funding.** This work was supported by National Institutes of Health grants R01-DK45641 to MJT, R01-HD17379 to MJT, R01-HL076448 to SYT, P01-DK59820 to SYT and MJT, U54-HD007495 to FJD and SYT as well as by R01-CA77530 to JPL. Ligand assay was supported by National Institute of Child Health and Human Development (Specialized Cooperative Centers Program in Reproduction Research) grant U54-HD28934 to the University of Virginia Center for Research in Reproduction Ligand Assay and Analysis Core.

